# *BrRLP48*, Encoding a Receptor-Like Protein, Involved in Downy Mildew Resistance in *Brassica rapa*

**DOI:** 10.3389/fpls.2018.01708

**Published:** 2018-11-23

**Authors:** Bin Zhang, Pan Li, Tongbing Su, Peirong Li, Xiaoyun Xin, Weihong Wang, Xiuyun Zhao, Yangjun Yu, Deshuang Zhang, Shuancang Yu, Fenglan Zhang

**Affiliations:** ^1^Beijing Vegetable Research Center (BVRC), Beijing Academy of Agriculture and Forestry Sciences (BAAFS), Beijing, China; ^2^Key Laboratory of Biology and Genetic Improvement of Horticultural Crops (North China), Ministry of Agriculture and Rural Affairs, Beijing, China; ^3^Beijing Key Laboratory of Vegetable Germplasm Improvement, Beijing, China

**Keywords:** *Brassica rapa*, QTL, downy mildew, receptor-like protein, salicylic acid

## Abstract

Downy mildew, caused by *Hyaloperonospora parasitica*, is a major disease of *Brassica rapa* that causes large economic losses in many *B. rapa*-growing regions of the world. The genotype used in this study was based on a double haploid population derived from a cross between the Chinese cabbage line BY and a European turnip line MM, susceptible and resistant to downy mildew, respectively. We initially located a locus *Br-DM04* for downy mildew resistance in a region about 2.7 Mb on chromosome A04, which accounts for 22.3% of the phenotypic variation. Using a large F_2_ mapping population (1156 individuals) we further mapped *Br-DM04* within a 160 kb region, containing 17 genes encoding proteins. Based on sequence annotations for these genes, four candidate genes related to disease resistance, *BrLRR1, BrLRR2, BrRLP47*, and *BrRLP48* were identified. Overexpression of both *BrRLP47* and *BrRLP48* using a transient expression system significantly enhanced the downy mildew resistance of the susceptible line BY. But only the leaves infiltrated with RNAi construct of *BrRLP48* could significantly reduce the disease resistance in resistant line MM. Furthermore, promoter sequence analysis showed that one salicylic acid (SA) and two jasmonic acid-responsive transcript elements were found in *BrRLP48* from the resistant line, but not in the susceptible one. Real-time PCR analysis showed that the expression level of *BrRLP48* was significantly induced by inoculation with downy mildew or SA treatment in the resistant line MM. Based on these findings, we concluded that *BrRLP48* was involved in disease resistant response and the disease-inducible expression of *BrRLP48* contributed to the downy mildew resistance. These findings led to a new understanding of the mechanisms of resistance and lay the foundation for marker-assisted selection to improve downy mildew resistance in *Brassica rapa*.

## Introduction

*Brassica rapa*, a species in the Cruciferae family, has great economic value. Some vegetable forms of *B. rapa* mainly include Chinese cabbage, pak choi, and turnip. In particular, Chinese cabbage (*B. rapa* L. ssp. *pekinensis*) is one of the most important vegetable crops in China with the highest growth area and yield among cultivated vegetables (Liu X.M. et al., 2010). However, Chinese cabbage is susceptible to downy mildew disease caused by the oomycetes *Hyaloperonospora parasitica var brassicae* which tends to propagate under warm and high humidity conditions in spring and autumn, which results in up to 90% yield loss (Göker et al., 2009; Yu et al., 2009). Seedlings are most susceptible. However, the plants can be damaged during the whole growth period. Frequent fungicide application is essential to control the infection, but this severely endangers food safety and sustainable agriculture development. Therefore, the identification of resistant germplasm resources and the breeding of disease-resistant varieties of *B. rapa* are indispensable to reduce the yield loss caused by Brassica downy mildew.

Quantitative trait loci (QTL) mapping combined with marker-associated breeding has been one of the most effective methods to find disease resistance genes and create disease-resistant varieties. Genetic analysis of downy mildew resistance in Chinese cabbage firstly initiated several decades before showed that the resistance was determined by a single dominant gene (Niu et al., 1983). Yu et al. (2009) demonstrated that the downy mildew resistance is a trait with high heritability in seedling stage of Chinese cabbage and controlled by a major dominant gene as well as some minor modifying genes. A major QTL, effective at every developmental stage, was detected on linkage group A08 by both interval and MQM mapping methods (Yu et al., 2009, 2016). A single dominant locus designated *BrRHP1* was located in a 10.5-CM region on A01 Chromosome at the heading stage of Chinese cabbage (Kim et al., 2011). In addition, two minor QTLs, located on linkage groups A06 and A04 were effective at the rosette and heading stages (Yu et al., 2016). The resistance of different natural variations was probably conferred mainly by different genes involving in resistant response. The genetic resistance to downy mildew of plants in different growth stages was also somewhat different (Yu et al., 2016). Based on next-generation sequencing technology, specific-locus amplified fragment sequencing (SLAF-seq) was developed for mapping traits of interest, with the advantages of being a technique of high-throughput, high accuracy, low cost, and short time (Wang et al., 2012; Chen et al., 2013; Sun et al., 2013; Zhang et al., 2013). An improved SLAF-seq strategy was utilized to construct a high-density bin-map, providing abundant developed single-nucleotide polymorphisms (SNPs) markers which could be used to locate QTLs for resistance to downy mildew (Yu et al., 2016). Genome wide re-sequencing was used to obtain the sequences of different individuals based on reference genomic sequences. On account of the sequence variance analysis of different individuals, plenty of high density and high quality SNPs and InDel (insertion/deletion) markers were found which would be efficient tools to assist in QTL fine mapping.

Plants possess an innate immune system, which includes signal recognition and resistance responses, to defend pathogens. This occurs under strong evolutionary pressures coming from significant constraints on the plant’s fitness caused by pathogens. Immune receptors mediate recognition of molecules derived from pathogens or from the damages caused by them (Cook et al., 2015). Nucleotide-binding, leucine-rich repeat (NB-LRR) proteins, highly similar to NOD-like receptors (NLRs) in animals, were identified as the first major group of plant immune receptors, encoded by classical plant disease resistance (*R*) genes (Bent et al., 1994; Grant et al., 1995; Salmeron et al., 1996; Parker et al., 1997; Botella et al., 1998; Warren et al., 1998). In subsequent research, the *R* genes that were cloned not only encoded NB-LRR proteins, but also encoded receptor-like proteins (RLPs), consisting of an extracellular LRR and transmembrane (TM) domain, as well as receptor-like kinases (RLKs), including an extracellular LRR, TM domain, and a cytoplasmic kinase domain (Verica and He, 2002). Several *RLP* genes functioning in disease resistance were first identified in tomato, such as *Cf-2, Cf-4, Cf-5*, and *Cf-9* (Jones et al., 1994; Dixon et al., 1996; Joosten et al., 1997; Thomas et al., 1997; Dixon et al., 1998; Takken et al., 1999; Panter et al., 2002). In the last few decades, an increasing number of *RLPs* was cloned as immune receptors in plant disease resistance. For example, *RPP27* for *Arabidopsis* downy mildew resistance, *Ve1* and *LeEix1* in tomato, and *LepR3* involved in blackleg resistance in *Brassica napus* (Bar et al., 2010; de Jonge et al., 2012; Larkan et al., 2013). In particular, extracellular LRR domains in RLPs may play a key role in pathogen recognition. For instance, Cf-4 has two less LRR domains than Cf-9, which leads to differences in resistance responses (van der Hoorn et al., 2001). The first 30 LRRs of Ve1 were required for Ve1-mediated *Verticillium* resistance in tomato, deletion of LRRs resulted in loss of functionality (Fradin et al., 2014). In addition, the absence of cytoplasmic kinase domains in RLPs compared with RLKs has enabled RLPs to interact with other RLKs to activate cytoplasmic signaling pathways responsive to pathogen invasion. *Solanum lycopersicum* LRR RLK (SOBIR1) and BRI1-associated receptor kinase 1 (BAK1) were shown to form a complex with Cf-type proteins (Cf-2, Cf-4, Cf-9) and LeEix1 to mediate a disease resistance response (Bar et al., 2010; Liebrand et al., 2013; Zhang et al., 2013; Liebrand et al., 2014).

Phytohormones play pivotal roles in the defense regulation network, by which plants can translate the pathogen-induced early signaling events into activation of effective defense responses (Pieterse et al., 2009). Among these, jasmonic acid (JA) and salicylic acid (SA) are considered to be the major defense-related phytohormones, in addition to other phytohormones such as ethylene (ET), abscisic acid (ABA), auxin, gibberellins (GAs), cytokinins (CK), and brassinosteroids (BRs) (Shigenaga and Argueso, 2016). Previous studies showed that there is a severe alteration in the level of resistance to specific types of pathogens, depending on the phytohormone involved. SA has been shown to regulate the defense responses triggered by biotrophic pathogens (Thomma et al., 2001). After pathogen infection, increased SA levels both in local and distal parts of plants activates defense responses through nonexpressor of pathogenesis-related genes 1 (NPR1), a component downstream of SA. NPR1 translocates to the nucleus to interact with TGA-type transcription factors (TGA2, TGA5, and TGA6) to initiate the expression of SA-dependent genes including *PR* genes (Kinkema et al., 2000; Zhang et al., 2003; Dong, 2004). Previous studies have demonstrated that WRKY transcription factors and NPR1 exhibit a synergistic effect on the regulation of the expression of *PR* genes (Mou et al., 2003; Xie et al., 2010).

In contrast, JA biosynthesis and signaling are required for resistance to necrotrophic pathogens (Glazebrook, 2005). In JA-mediated signaling, coronatine-insensitive 1 (COI1) was proven to be responsible for signal perception and releasing a group of MYC transcription factors to initiate transcriptional reprogramming, by mediating 26S proteasome-dependent degradation of the JAZ (JA ZIM-domain) family of proteins, which act as transcriptional repressors (Wasternack and Hause, 2013). In both SA and JA signal transduction, the transcriptions of genes involved in defense response were significantly reprogrammed. But the transcription of *R* genes regulated by hormones involved in resistance responses has been not reported.

In this study, we identified candidate downy mildew resistance genes encoding LRR-containing proteins in *Br*-*DM04*, within a 160-kb region. Using a transient expression system, overexpression of *BrRLP48* could enhance resistance to downy mildew in a susceptible line. Otherwise, the leaves infiltrated with RNAi construct of *BrRLP48* could reduce the disease resistance of resistant parent MM. Subsequently, through sequence and expression analysis after downy mildew infection, we found that *BrRLP48* was significantly induced in resistant line probably due to the presence of the putative SA and JA responsive elements in the promoter. Finally, the content of SA in the resistant line MM was dramatically elevated after inoculation, with only the expression of *BrRLP48* significantly induced under SA treatment, correlating with an increased expression following pathogen invasion. These results demonstrated the involvement of *R* genes in Brassica downy mildew resistance and shed light on the molecular mechanism for marker-assisted breeding of disease-resistant varieties in *B. rapa*.

## Materials and Methods

### Plant Materials

A mapping population was derived from a cross between BY and MM. BY is a Chinese cabbage in-bred line (*B. rapa* ssp. *pekinensis*) that was self-fertilized for eight generations. MM is a European turnip line (Manchester Market; *B. rapa* L. ssp. *rapifera*) that was self-fertilized for six generations. The resulting F1 plants were subjected to self-pollination and microspore culture to produce an F_2_ segregating population and a double haploid (DH) population with 80 DH lines. The method of embryogenesis and doubling by isolated microspore culture in Chinese cabbage was described in Wang et al. (2010).

### Downy Mildew Inoculation and Evaluation of Resistance and Susceptible Phenotypes in the Mapping Populations

The seeds of the F_2_ and DH mapping population were germinated on wet filter paper at 25°C for 24***–***48 h. The seedlings were then transferred into soil and were grown at 25°C with 16 h light followed by 20°C in an 8 h dark cycle. For the DH population, three inoculation replicates were conducted, with 10 plants per replicate (*n* = 30). Two week-old seedlings were used for downy mildew inoculation. The pathogen isolate used in this study and the method of inoculum maintained are described in Yu et al. (2009). As described previously (Yu et al., 2009), about 2 × 10^5^ spores μL^-1^ of conidial suspension of the *P. parasitica* isolate were sprayed on the abaxial side of leaves. After inoculation, the seedlings needed dark conditions and high humidity (nearly 100%) during the first 24 h and high humidity with 16 h light/8 h dark for subsequent 3***–***4 days for pathogen invasion. Inoculated plants were grown under 100% humidity and dark conditions at 20°C for 24 h. After 1 week high humidity treatment, phenotypic data were collected. For the F_2_ population, the susceptible phenotype was evaluated according to the rating system described by Yu et al. (2009). Plants in classes 0, 1, and 3 with no sporulation observed were considered resistant (R), and those in classes 5, 7, and 9 with obvious sporulation were classified as susceptible (S). For the DH population, the disease indices (DIs) for each line were calculated as described in Yu et al. (2009). For further molecular marker analysis, young leaves were collected and DNA was subsequently extracted as described by Murray and Thompson (1980), with minor modifications.

### Molecular Marker Analysis

Genomic DNA of the parental lines was re-sequenced by BioMarker Co. (Beijing) and the results were compared to the reference sequence of *Chiifu-401-42* (v 1.5). Sequence differences between the parental line in the target QTL region were used to develop SNP markers, designed and synthesized in Britain by the Laboratory of the Government Chemist (LGC) Co., Ltd., for genotyping using the Kompetitive Allele-Specific PCR (KASP) method, as described by Saxena et al. (2014). For each SNP, two allele-specific forward primers and one common reverse primer were designed and tested by LGC. The PCR reactions of 20 μL contained 2 μL PCR buffer, 0.8 μL dNTPs (2.5 mM each), 1.0 μL oligonucleotide primers each (10 μM), 0.2 μL Taq DNA polymerase (2.5 U/μL), 2 μL DNA template (50 ng/μL), and 13 μL ddH_2_O. The amplifications were performed in a PTC-200 Thermal-cycler (Bio-Rad Laboratories, Hercules, CA, United States) using the following program: 95°C for 5 min followed by 35 cycles of 95°C for 30 s, 55°C for 30 s, and 72°C for 30 s, with a final extension at 72°C for 5 min. Fluorescent detection of the reaction products was performed using an Omega Fluorostar scanner (BMG LABTECH GmbH, Offenburg, Germany), and the data were analyzed using KlusterCaller 1.1 software (KBioscience). A total of 14 SNP markers were screened to combine with the phenotypes of F_2_ population and locate the *Br*-*DM04* to a 160-kb region.

### Linkage Map Construction and QTL Analysis

The linkage map was constructed with JoinMap 4.0 software (Van Ooijen, 2006). The details of linkage map construction were identical to Su et al. (2018). The Kosambi mapping function was used to calculate genetic linkage distances (Kosambi, 1944). Logarithm of odds difference (LOD) thresholds for determining significant QTLs were estimated from 1000 permutation tests (*P* < 0.05; Churchill and Doerge, 1994). The genome-wide threshold for downy mildew resistance was 3.0, and interval mapping was used to identify putative QTLs (Lander and Botstein, 1989; Young, 1996). Subsequently, molecular markers coinciding with, or closely flanking, the maximum QTL LOD were used as co-factors in multiple QTL analysis. All calculations employed MapQTL 3.0 software (Van Ooijen et al., 2002). In order to provide visual images of marker loci genomic positions, integrated markers and QTL were plotted using Mapchart 2.2 (Voorrips, 2002).

### Gene Cloning and DNA Sequence Analysis

The primers used for amplification of genomic DNA sequences of *BrRLP47, BrRLP48, BrLRR1*, and *BrLRR2* were designed using reference sequences identified from the Brassica database^1^. The PCR products were then cloned into the pCR8/GM/TOPO^TM^ entry vector (Invitrogen, United States) and sequenced. Sequence similarities were calculated using DNAMAN version 6. The coding sequences of *BrRLP47, BrRLP48, BrLRR1*, and *BrLRR2* were cut by using Hind III and Kpn I restriction sites at 5′- and 3′-terminals, respectively (BGI, Beijing, China). Subsequently, they were cloned into the *pSuper1300* vector fused with a GFP tag at the C-terminus (Gong et al., 2002). All the primers used are listed in Supplementary Table S4.

The pYBA1311 vector was used for the RNA interference (RNAi) analysis (Yan et al., 2012). In *BrRLP47* CDS, a highly specific fragment containing 169 bp was amplified with two pairs of adaptors (*Kpn* I/*Xho* I and *Cla* I/*Bam*H I). To generate hairpin RNA, the fragment was inserted into *Kpn* I/*Xho* I sites and *Cla* I/*Bam*H I sites of pYBA1311 to form a pair of reverse complement sequences along the transcript direction. A 153 bp fragment in the CDS sequence of *BrRLP48* was constructed into pYBA1311 vector in the same way. Then these recombinant plasmids were used to transform into *Agrobacterium tumefaciens* strain GV3101 for further agroinfiltration. The primer sequences used were listed in Supplementary Table S4.

### RNA Extraction and Real-Time PCR Analysis

Total RNA was isolated from leaves with the plant RNeasy kit (TIANGEN, Beijing, China). RNA was reverse transcribed into cDNA using a PrimeScript^TM^ RT Reagent Kit (TaKaRa, Osaka, Japan). Real-time PCR reactions were performed using the SYBR Green I Master Mix (Roche, Basel, Switzerland) and were quantified with the Light Cycler 480 II (Roche, Basel, Switzerland). Reaction conditions were as follows: an initial denaturation at 95°C for 3 min, followed by 40 cycles of 95°C for 15 s (denaturation), 60°C for 30 s (annealing), and 72°C for 45 s (extension). PCR amplification was followed by heating for 1 min at 60–95°C for melting curve analysis. Each sample reaction was performed with three replicates using 5 μL of Master Mix, 0.25 μM of each primer, 1 μL of diluted cDNA, and DNase-free water to a final volume of 10 μL. The PCR products were sequenced to confirm the gene-specific amplifications.

### Expression Analysis of Candidate-Resistant Genes After Inoculation

Two-week old seedlings of disease resistant and susceptible parents MM and BY were used for downy mildew inoculation. Thirty plants of MM and BY were used (*n* = 30). Under the same high humidity condition, the leaves with inoculated and non-inoculated leaves were sampled at 0, 2, 6, 12, 24, and 48 h after inoculation. Ten to fifteen leaves were mixed to one sample. Then RNA of these samples was extracted for qRT-PCR analysis. Three technical replicates were performed. The primers of *BrRLP47, BrRLP48, BrLRR1*, and *BrLRR2* for qRT-PCR analysis were designed by Primer Premier 5 (Lalitha, 2000) and listed in Supplementary Table S4.

### Agroinfiltration

*Agrobacterium tumefaciens* strain GV3101 was used for transient expression studies. The cotyledons of 7-day old seedlings were used in the Agroinfiltration experiment. Methodology was employed following Liu L.J. et al. (2010) with minor modifications. The Agrobactierium strains including gene-silencing supressor p19 were grown at 28°C to an OD_600_ of approximately 2.5. Then the bacteria pellets were resuspended in 10 mM MgCl_2_. The p19 and each strain were mixed with to final OD_600_ of 0.1 and 0.2. Final concentration of 10 mM Acetosyringone and 200 μM 2-(*N*-morpholine)-ethanesulfonic acid (MES, pH 5.6) were added to the mixture, which was kept at room temperature for at least 3 h without shaking before infiltration. Cotyledon injection was performed by depressing a 2-mL syringe to the back of leaf till the mixture was infiltrated to the whole leaf. Cotyledons without injection and with empty vector infiltrated were conducted as the controls. Overexpression vector with GFP tag was infiltrated into the cotyledons of susceptible parent BY and RNAi vector was injected into the cotyledons of resistant parent MM. Three days after infiltration, GFP fluorescence was observed under the confocal microscope (Zeiss LSM700, Japan) and 5***–***10 injected cotyledons were mixed to each sample for qRT-PCR analysis. The RNA samples in three biological replicates were employed for qRT-PCR analysis. Subsequently, downy mildew was inoculated into these 3-day infiltrated cotyledons. Keeping high humidity for 5***–***7 days after inoculation, the disease-resistant phenotype of the infiltrated cotyledons was investigated. The infected cotyledon, of which more than a half leaf area with observed sporulation, was considered to be the susceptible cotyledon. The expression levels of injected genes and the percents of susceptible cotyledons were derived from four independent experiments.

### SA and JA Treatments

Eighteen-day-old BY and MM seedlings with two or three fully expanded leaves were subjected to SA and JA treatments. SA at 1.5 mM (Sigma-Aldrich, Lot V900072) and 1 mM Me-JA (Sigma-Aldrich, Lot 392707) dissolved in water were sprayed on the abaxial side of the leaves. Seedlings sprayed with water were designated as the controls. The leaves were sampled at 0, 2, 4, 6, 8, 12, 24, 48, 72, 96, and 120 h for detection of gene expression. Five to eight leaves were pooled in each sample. Three parallel experiments were performed as the biological replicates in further qRT-PCR analysis. The ratio of gene expression under phytohormone treatment to that under water treatment was considered to be the gene induction level by the phytohormone.

### Phytohormone Measurement

The leaves of 20-day old BY and MM seedlings were placed under high humidity and dark condition for 6 h and then downy mildew inoculation was performed. The samples were collected at 0, 4, and 12 h after downy mildew inoculation (the inoculation method was described in the section “Downy mildew inoculation and evaluation of resistance and susceptible phenotypes in the mapping populations”). For each sample for SA and JA measurements, approximately 50 mg of leaf tissue from 5 to 10 seedlings was needed. Three biological repetitions were made at each time point, consisting each one on a mix of five seedlings. After freezing in liquid nitrogen, the samples were stored at -80°C until further analysis. Each sample was ground to a fine powder in liquid nitrogen. The 50 mg powder was extracted in 15 mL cold 100% methanol followed by sonication in ultrasonic water bath (MRC, Holon, Israel) for 30 min at frequency 40 kHz (25°C). Then the mixture was centrifuged at 18,000 × *g* for 10 min at 25°C. The supernatant was filtered through 0.2-μm membrane and dried under vacuum condition. The residue was dissolved in 400 μL of H_2_O/methanol (1:1, v/v). Samples were centrifuged (10 min at 18,000 × *g*), and 10 μL of the aqueous phase was retained for analysis. The external standard method was used for quantification, in which internal standard SA (CAS: 69-72-7) and JA (CAS: 3572-66-5) were diluted in methanol to the concentrations of 1, 2, 5, 10, 20, 50, and 100 ng/mL to draw standard curve. The contents of free SA and JA were measured using a Shimadzu UHPLC system (Kyoto, Japan) equipped a LC-30AD solvent delivery system, a SIL-30AC autosampler, a CTO-30A column oven, a DGU-20A3 degasser, and a CBM-20A controller (Fan-Xing Biological Technology- Beijing Co., Ltd.).

## Results

### Genetic Mapping of Bra-DM

**FIGURE 1:**
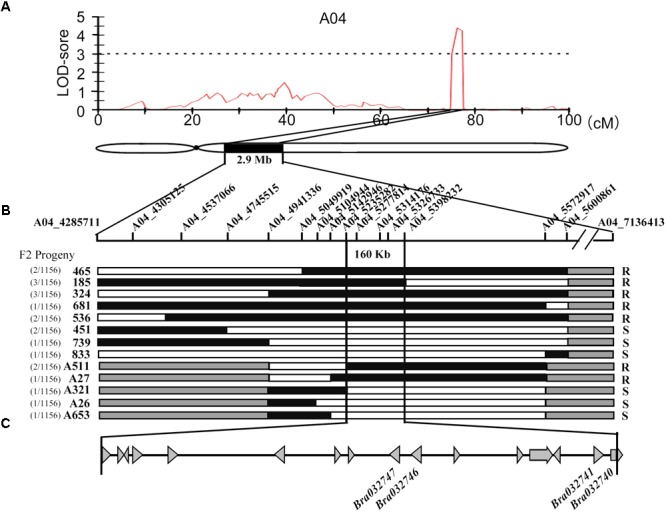
Fine mapping of the downy mildew resistant QTL Bra-DM04. **(A)** Major QTL likelihood maps by multiple QTL model (MQM). The LOD-score threshold of 3.1 is indicated by the dashed line. The highest LOD score of 4.2 was located in a 2.75 cM on A04 Chromosome between SNP marker A04_4285711 and A04_7136413. Below the likelihood map was the schematic diagram of Chromosome A04, on which a 2.9-Mb region corresponding to the 2.75 cM region in likelihood map was filled with black. **(B)** F_2_ population was applied to fine mapping of *Bra*-*DM04*, narrowing down the area to 160 kb. The SNP markers used for mapping are labeled on a schematic diagram of the chromosome. The representative genotypes of recombinants were below the chromosome. The numbers of recombinants of the same genotype and the total number of population are written on the left of the recombinants’ names. Phenotypes are on the right. “R” and “S” refer to resistant and susceptible, respectively. Black and white rectangles represent resistant and susceptible genotypes, respectively. Gray segments indicate regions that were not tested with markers. **(C)** The gray triangles denote the genes in *Bra*-*DM04*. This region contains 16 candidate genes, among which four predicted resistance genes are indicated.

To locate the major QTL for downy mildew resistance, we used the DH mapping population derived from a cross between the Chinese cabbage line BY (*B. rapa* ssp. *pekinensis*), and a European turnip line MM (*B. rapa L.* ssp. *rapifera*). A sequence-based bin map was constructed using an improved specific length amplified fragment sequencing (SLAF-seq) strategy (Su et al., 2018). The final bin map included 974 bins on the 10 linkage groups and was 1033.24 cM in length with an average distance of 1.06 cM between adjacent markers. After pathogen invasion, the leaves in resistant lines activated the hypersensitive response (HR)-based resistance to downy mildew causing the observed host necrosis but no sporulation. The sporulation could be observed on susceptible leaves. The disease susceptible parent of DH mapping population, BY is highly susceptible to downy mildew with a DI of 79.0. The resistant parent used in this work, MM, exhibited the prominent resistance to downy mildew (DI = 12.4) and the genetic basis of its resistance was worth for further investigation. The downy mildew-resistant phenotypic distribution of 77 DH individuals was showed in Supplementary Figure 1. The nearly complete resistance (DI = 7.0) of F1 progeny suggested that the disease-resistant phenotype was controlled by dominant-resistant gene in the population. Eventually, a resistant QTL, *Br*-*DM04* was roughly mapped to a long arm of chromosome A04 in a 2.75-cM region (from 74.58 to 77.33 cM) between SNP markers A04_4285711 and A04_7136911, with maximum LOD value 4.2, which explained up to 22.3% of the phenotypic variance (Figure 1A and Supplementary Figure 2).

To fine map *Br*-*DM04*, an F_2_ population with a total of 1156 individuals derived from the cross between BY and MM was employed to analyze disease-resistant phenotypes (Supplementary Figure 3). The rating system of susceptible phenotype was used in disease resistant investigation. F_2_ individuals in classes 0, 1, and 3 with no sporulation observed were considered resistant (R), and those in classes 5, 7, and 9 with obvious sporulation were classified as susceptible (S). We acquired 48,334 SNP markers on Chromosome A04 based on resequencing results and 5,339 SNP markers in initially located regions were employed to make the markers more dense. Fourteen of 6,169 SNPs which showed polymorphisms were selected for Kompetitive Allele-Specific PCR (KASP) assay development and used to screen the F_2_ population to identify recombinants (Figure 1B and Supplementary Table S1). Finally, the Brassica downy mildew resistant QTL, *Br*-*DM04*, was located in a 160-kb region, between the SNP markers A04_5235282 and A04_5398232 (Figure 1B). Specially, the SNP marker within the gene *Bra032747*, A04_5326733, co-segregated with downy mildew resistance. In addition, we sequenced the five markers closely linked to *Br*-*DM04* and found that the order of the markers was consistent with the order of their homologs in Chiifu-401 of *B. rapa* (Wang et al., 2011).

### Identification and Coding Sequences Analysis of Candidate Disease Resistance Genes

To identify the candidate resistance genes in *Br*-*DM04*, a comparative gene annotation in *Arabidopsis thaliana* was investigated. There are 17 genes located in *Br*-*DM04*, in which 16 have functional annotations referring to their *Arabidopsis* homologs (Figure 1C and Table 1). Among these genes, we first focused on the *R* genes. Therefore, according to the annotations in Table 1, there are only four gene candidates: *Bra032747, Bra032746, Bra032741*, and *Bra032740*, probably functioned in disease resistance. *Bra032747* and *Bra03276* encode RLPs containing seven and nine LRRs, respectively. Thus, *Bra032747* and *Bra03276* were renamed as *BrRLP48* and *BrRLP47*, according to their homologous genes, *AtRLP48* and *AtRLP47*, in *Arabidopsis* (Table 1). *Bra032740* and *Bra032741*, whose homologous genes in *Arabidopsis* were assigned as the disease resistance gene family, belong to the LRR gene family and encode proteins containing six LRR motifs, but lack a TM domain, compared with RLPs. *Bra032740* and *Bra032741* were named as *BrLRR1* and *BrLRR2*. The genes encoding RLP and LRR families of proteins are known as two types of resistance genes (*R* genes) involved in plant disease resistance (Jones et al., 1994; Song et al., 1995; Dixon et al., 1996, 1998; Joosten et al., 1997; Thomas et al., 1997; Takken et al., 1999; Panter et al., 2002). Therefore, *BrRLP48, BrRLP47, BrLRR1*, and *BrLRR2* were identified as candidate genes for downy mildew resistance.

**Table 1:** Sixteen annotated genes in the 160 kb region of *Bra-DM04*.

Gene ID	Chr	Start	Stop	Homologous gene in *Arabidopsis*	Annotations in *Arabidopsis*
Bra032756	A04	5186044	5188004	AT4G14040	EDA38, SBP2; SBP2 (selenium-binding protein 2); selenium binding
Bra032755	A04	5223891	5226212	AT4G14030	SBP1; SBP1 (selenium-binding protein 1); selenium binding
Bra032754	A04	5229364	5229723	AT4G14010	RALFL32; RALFL32 (ralf-like 32); signal transducer
Bra032753	A04	5230263	5231737	AT4G14000	Unknown protein
Bra032752	A04	5234243	5235756	AT4G13980	AT-HSFA5, HSFA5; AT-HSFA5; DNA binding/transcription factor
Bra032751	A04	5246918	5249840	AT4G13970	Zinc ion binding
Bra032750	A04	5286031	5287637	AT4G13940	HOG1, EMB1395, SAHH1, MEE58 (maternal effect embryo arrest 58)
Bra032749	A04	5305170	5307173	AT4G13930	SHM4 (serine hydroxymethyltransferase 4)
Bra032748	A04	5310018	5311786	AT4G13870	WEX, ATWEX; WRNEXO (Werner syndrome-like exonuclease)
Bra032747	A04	5325435	5328188	AT4G13880	AtRLP48; AtRLP48 (receptor-like protein 48); protein binding
Bra032746	A04	5333196	5335784	AT4G13810	AtRLP47; AtRLP47 (receptor-like protein 47); protein binding
Bra032745	A04	5347152	5347692	AT1G52950	Replication protein-related
Bra032744	A04	5369216	5371600	AT3G54010	PAS1, DEI1; PAS1 (PASTICCINO 1); FK506 binding/binding/peptidyl-prolyl *cis*–*trans* isomerase
Bra032743	A04	5374068	5381967	AT4G13840	Transferase family protein
Bra032742	A04	5382581	5383837	AT4G13830	J20; J20 (DNAJ-LIKE 20); heat shock protein binding
Bra032741	A04	5396373	5399403	AT4G13820	Disease resistance family protein/LRR family protein
Bra032740	A04	5402699	5406970	AT4G13820	Disease resistance family protein/LRR family protein

To characterize the disease resistance function of the candidate genes, sequence analysis of the coding region in disease-resistant and susceptible parents, MM and BY, respectively, was initially performed. We designed the primers according to the reference sequence of *B. rapa* (*Chiifu-401-42* v1.5). The genomic sequences of *BrRLP47* and *BrRLP48* are approximately 2.5 kb based on the reference sequence, and *BrLRR1* and *BrLRR2* are 4.2 and 3 kb, respectively. Fractional amplification by PCR was employed to obtain the complete sequences of *BrLRR1* and *BrLRR2* due to their sequence complexity and excessive length. The sequences of *BrRLP47, BrRLP48, BrLRR1*, and *BrLRR2* in the susceptible parent BY are completely consistent with reference sequences (Supplementary Figure 4). The genomic sequence of *BrLRR1* in the resistant line MM had only two single nucleotide variations (Supplementary Figure 4). There is a 56 bp insertion, a 24 bp deletion, and a 89 bp deletion in the *BrLRR2, BrRLP47*, and *BrRLP48* sequences, respectively, except for single nucleotide variations (Supplementary Figures 4, 5A). However, the predicted protein domains showed no distinction in four candidate genes between resistant (MM) and susceptible parents (BY) (Supplementary Figure 5B).

### Overexpression and Knockdown of Candidate Genes in Transient Expression System

**FIGURE 2:**
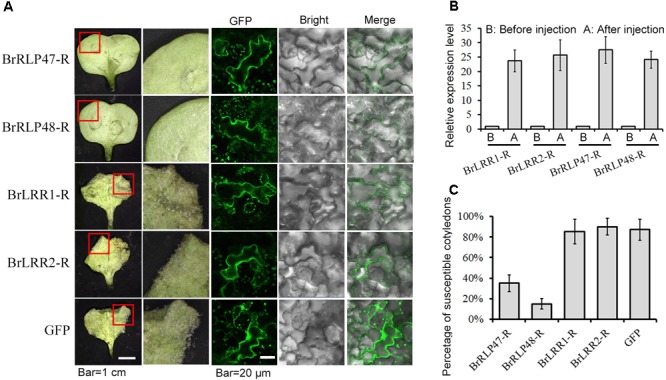
Functional analysis of resistance candidate genes in a transient expression system mediated by agroinfiltration. **(A)** The disease resistance phenotypes and protein expression assay of cotyledons injected with *BrLRR1-GFP, BrLRR2-GFP, BrRLP47-GFP, BrRLP48-GFP*, and *GFP* alone were observed 3 days after agroinfiltration. The images in red squares are magnified in the pictures in the second column from the left. **(B)** Gene expression analysis after agroinfiltration. Five to ten leaves were pooled in a single sample in each replicate. Expression of each gene before injection was defined as 1.0. Values represent means ±*SD* (*n* = 3) from three biological replicates. **(C)** The percentage of susceptible injected cotyledons after inoculation. Values represent means ± *SD* from four independent experiments. Each experiment tested at least 50 cotyledons.

To identify which candidate gene confers resistance to downy mildew, we developed an *Agrobacterium*-mediated transient expression system in cotyledons of *B. rapa* for functional analysis, instead of using time-consuming transgenic methods. The disease resistant functions of these four candidate genes from resistant parent MM (*BrLRR1-R, BrLRR2-R, BrRLP47-R*, and *BrRLP48-R*) were firstly detected. The sequences of *BrLRR1-R, BrLRR2-R, BrRLP47-R*, and *BrRLP48-R* were cloned into the vector *pSuper1300* containing a C-terminal GFP tag. The *Agrobacterium* carrying these vectors were injected into the cotyledons of 7-day-old seedlings of the susceptible parent BY, with the empty vector as a negative control. Three days after infiltration, GFP fluorescence was detected in infiltrated cotyledons. Using confocal microscopy with the same parameters, similar fluorescence intensity was observed, indicating similar protein expression levels between four candidate genes and *GFP* in the empty vector (Figure 2A). In the meantime, the expression levels of these genes were enhanced approximately 23.7–27.5-fold, compared with controls before infiltration (Figure 2B). Subsequently, downy mildew was inoculated into 3-day infiltrated cotyledons. The susceptible cotyledons could easily have sporulation observed rarely with the host necrosis being seen. Therefore, for the phenotype investigation, a cotyledon, more than half infiltrated area had sporulation observed, was defined as a susceptible cotyledon. Cotyledons of seedlings with different growth conditions could directly influence the disease resistant phenotype. Thus, we calculated the percentage of susceptible cotyledons to reflect the disease-susceptible phenotype. With similar expression levels, the disease resistance of cotyledons transiently expressing *BrRLP47* and *BrRLP48* was significantly elevated compared to control; there were only 35.5 ± 8.1 and 15.8 ± 5.6% susceptible cotyledons compared with 87.4 ± 10.7% in control (Figure 2C). However, the susceptibility of BY did not change with the transient expression of *BrLRR1* and *BrLRR2*, with the percentage of susceptible cotyledons being 85.3 ± 12.1 and 90.2 ± 8.8%, respectively (Figure 2). Furthermore, we overexpressed the alleles of *BrRLP47* and *BrRLP48* (up to 25.1 and 27.9-fold) from susceptible parent BY (*BrRLP47-S* and *BrRLP48-S*), and we found they could also increase the disease resistance of BY (with the percentage of susceptible cotyledons being 33.1 ± 6.2 and 25.3 ± 3.4%, respectively) (Supplementary Figure 6). As a result, *BrRLP47* and *BrRLP48*, either from MM or BY, could enhance the resistance to downy mildew in the susceptible variety BY.

**FIGURE 3:**
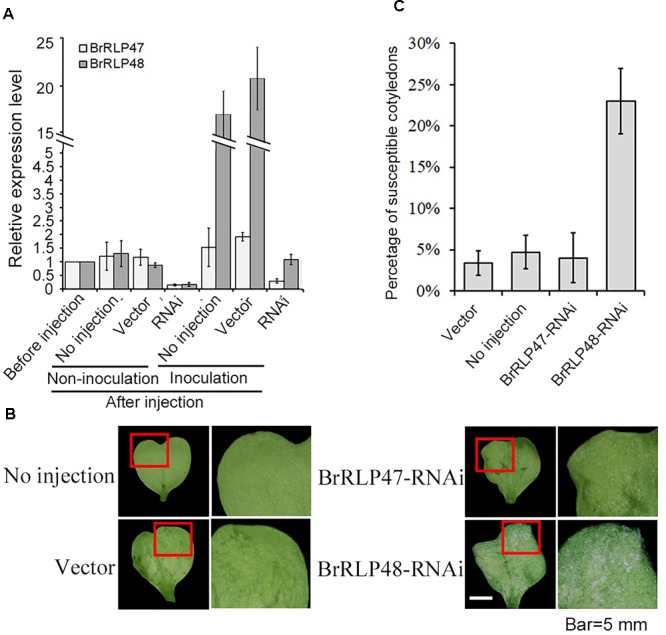
Functional analysis of resistance candidate genes in their knock-down lines through agroinfiltration-mediated transient expression system. **(A)** Gene expression analysis in the cotyledons infiltrated with RNAi construct of *BrRLP47* and *BrRLP48* after injection. The cotyledons before injection, without injection (no injection), and infiltrated with *Agrobacterium* carrying empty vector pYBA1311 (vector) were conducted as controls. Three days after agroinfiltration without inoculation, the gene expressions were detected in “non-inoculation” group. The gene expressions in infiltrated cotyledons at 6 h after inoculation were tested in “Inoculation” group. Five to ten leaves were pooled in a single sample in each replicate. Expression of each gene in the cotyledons before injection was defined as 1.0. Values represent means ± *SD* (*n* = 3) from three biological replicates. **(B)** The disease susceptible phenotypes of cotyledons injected with *BrRLP47-RNAi, BrRLP48-RNAi, RNAi-empty vector (vector)*, and cotyledons without injection were observed 3 days after agroinfiltration. The images in red squares are magnified in the pictures in the second column from the left. **(C)** The percentage of susceptible injected cotyledons after inoculation. Values represent means ±*SD* from three independent experiments. Each experiment tested at least 50 cotyledons.

To further investigate the resistant functions of *BrRLP47* and *BrRLP48*, we constructed the RNAi vectors with a 169 and 153-bp fragment from *BrRLP47* and *BrRLP48*, respectively, and agro-infiltrated the cotyledons of resistant parent MM. The agroinfiltrated cotyledons of *BrRLP47* and *BrRLP48* showed varying degrees of expression inhibition compared to the non-injection control before or after inoculation (Figure 3A). We phenotypically characterized the RNAi cotyledons 4 days after downy mildew inoculation. The results showed that the cotyledons infiltrated with RNAi construct of *BrRLP48* were more susceptible than control plant, with a relatively large percentage of susceptible cotyledons (23.3 ± 4.1%) (Figures 3B,C). Above all, the phenotypes of overexpression and knockdown lines of *BrRLP48* suggested that *BrRLP48* probably functioned in the downy mildew resistant response.

### The Expression of Candidate Genes Following Downy Mildew Inoculation

**FIGURE 4:**
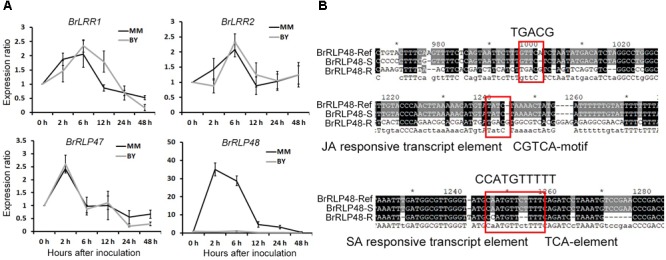
Expression analysis of resistance candidate genes after inoculation and promoter analysis of *BrRLP48* between BY and MM. **(A)** Expression analysis of *BrLRR1, BrLRR2, BrRLP47*, and *BrRLP48* after inoculation. Thirty seedlings of BY and MM were used. Ten to fifteen leaves were mixed in a sample. Expression of each gene at 0 h after inoculation was defined as 1.0. Values represent means ±*SD* (*n* = 3) from three technical replicates. **(B)** Predicted regulatory elements in the promoter of *BrRLP48* in MM. The sequences of MM in red rectangles are indicated above. “-R” and “-S” denote MM and BY, respectively.

The above results suggested that regulation of transcript levels of *BrRLP47* and *BrRLP48* may make plants exhibit different degrees of resistance to downy mildew. Therefore, we intended to investigate the expression levels of candidate genes under disease conditions and compared the promoter sequences of candidate genes between BY and MM. We collected the leaves of MM and BY after downy mildew infection for gene expression analysis through RT-PCR. Avoiding the high humidity condition, which was the pathogen invasion needed, to influence the gene expression, we sampled “0 h” samples under high humidity. Samples were taken from inoculated and non-inoculated leaves at 0, 2, 6, 12, 24, and 48 h after inoculation. The ratio of gene expression for inoculated to non-inoculated samples could reflect the induced expression level of genes following downy mildew inoculation. RT-PCR results showed that the expression of *BrLRR1* and *BrLRR2* was induced about twofold 6 h after inoculation and the induced patterns in MM were consistent with those in BY (Figure 4A). *BrRLP47* shared a similar gene expression pattern in MM and BY, elevated about 2.5- and 1.5-fold, respectively, at 2 h after inoculation (Figure 4A). The expression of *BrRLP48* was significantly stimulated 35-fold and 28-fold at 2 and 6 h, respectively, after inoculation in the resistant parent MM, but only 1.03-fold at 6 h in BY (Figure 4A). Thus, downy mildew invasion could significantly induce the expression of *BrRLP48* in the disease-resistant parent MM, which possibly conferred MM its disease resistance to downy mildew.

To further explore the reason why expression of *BrRLP48* was significantly increased in the resistant line MM after downy mildew inoculation, we first attempted to obtain the promoter sequences of *BrRLP48* in both resistant and susceptible parents. Based on reference sequences, we found that there were only 1,882 bp upstream of the ATG start codon in *BrRLP48*, including a 6 bp 5′-UTR region, the rest of which was considered as the promoter sequence. We designed the primers for ∼800–1,000 bp PCR products with pairwise overlap, according to the reference promoter sequence for amplification in both MM and BY. Finally, three fragments with pairwise overlap of 200–300 bp were obtained, which together assembled to 2,127 and 1,461 bp in MM and BY, respectively (Supplementary Figure 7). The 1,461 bp sequence in BY was completely consistent with the reference sequence (Supplementary Figure 7). Approximately 1,000 bp sequence upstream of the ATG start codon in MM was consistent with the reference sequence, containing several single nucleotide variations as well as multiple nucleotide insertions and deletions. But there were significant differences, including a 280 bp deletion and an 800 bp insertion in the rest of the promoter sequence (Supplementary Figure 7). The promoter sequences of *BrRLP48* in MM and BY, assigned as the resistant (R) and susceptible (S) promoters, respectively, were used for prediction of transcript elements using PlantCARE (a database of plant promoters and their *cis*-acting regulatory elements) (Higo et al., 1998). The transcript elements in R promoter sequences that did not exist in S promoters were those that were mostly involved in hormone responsiveness, including the auxin-responsive element TGA box, TCA element involved in SA responsiveness as well as TGACG and CGTCA motifs involved in MeJA (methyl-jasmonate) responsiveness (Figure 4B). SA and JA are the plant hormones that participate in disease resistance. Thus, the expression of *BrRLP48* could be regulated by the hormones involved in disease resistance after inoculation, possibly due to the hormone-responsive elements existing in R promoter sequences.

### The Concentration of SA and JA After Downy Mildew Inoculation

**FIGURE 5:**
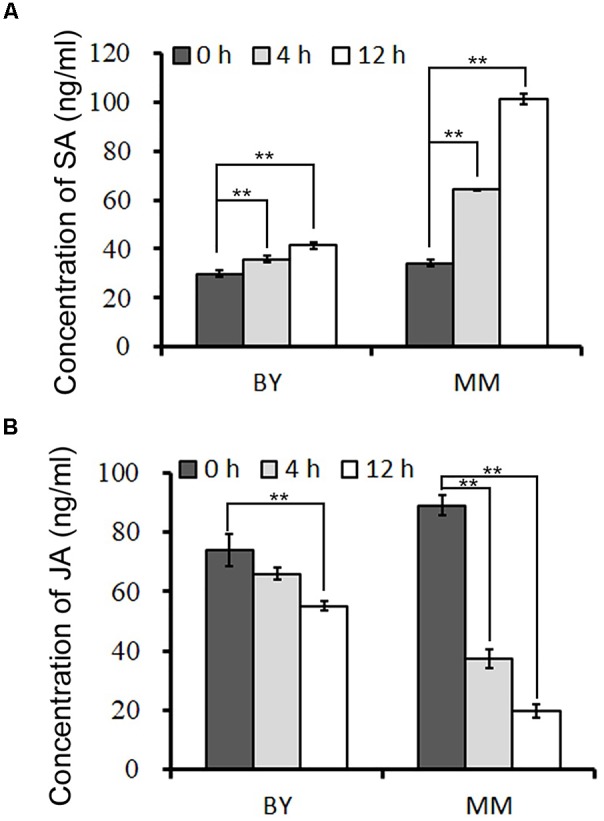
The concentrations of SA and JA after downy mildew inoculation. The concentrations of SA **(A)** and JA **(B)** in inoculated leaves were measured at 0, 4, and 12 h after downy mildew inoculation. The 50 mg leaves from 5 to 10 20-day old seedlings were collected in each sample. Values represent means ± *SD* (*n* = 3) from three biological replicates. Statistical significance between 0 and 4 or 12 h after inoculation in BY or MM was by a *t*-test: ^∗∗^*P* < 0.01.

To clarify which hormone-responsive element in the promoter of *BrRLP48* in MM responds to downy mildew, we first conducted experiments to identify the hormone involved in this process. The concentrations of SA and JA in resistant (MM) and susceptible (BY) parents during downy mildew infection were tested. The inoculated leaves were subjected to hormone detection analysis. The results showed that the concentrations of SA rose gradually at 4 and 12 h after inoculation in both BY and MM, while those of free JA declined (Figure 5 and Supplementary Table S2). Before inoculation, free SA was at similar concentrations in BY and MM, at 29.9 ± 1.2 and 34.3 ± 0.4 ng mL^-1^, respectively. Notably, the concentration of SA increased rapidly to 64.2 ± 0.1 and 101.3 ± 2.0 ng mL^-1^ at 4 and 12 h, respectively, after inoculation in MM; in contrast, the concentration peak in BY was 41.4 ± 1.5 ng mL^-1^ at 12 h after inoculation (Figure 5A and Supplementary Table S2). Thus, the free SA but not JA could significantly accumulate after inoculation in disease-resistant parent MM, indicating SA probably involved in downy mildew resistant response.

### The Expression of Candidate Genes Under SA and JA Treatment

**FIGURE 6:**
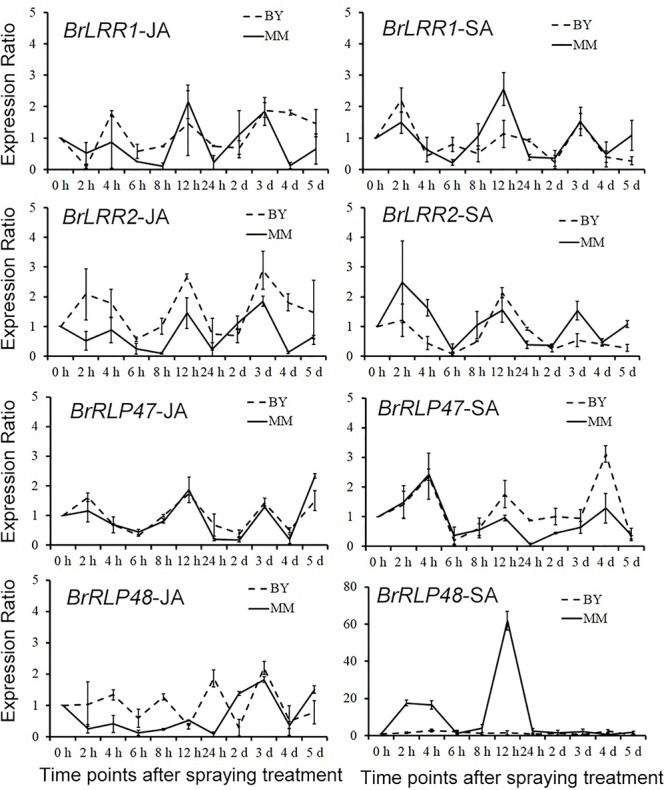
Expression analysis of candidate genes under SA and JA treatments. The numbers in *Y*-axis denote the ratios of expression levels under SA and JA treatments compared to the controls. Five to eight leaves were pooled in a single sample in each replicate. Expression of each gene at 0 h after SA or JA treatment in BY or MM was defined as 1.0. Values represent means ±*SD* (*n* = 3) from three biological replicates.

To confirm whether *BrRLP48* was induced by SA resulting from the SA-responsive element in MM, the expression analysis of four candidate resistance genes was performed after SA and JA treatment. SA (1.5 mM) and Me-JA (1 mM) were sprayed on the abaxial side of leaves of 14-day-old seedlings, with water used as a negative control. Sample collections were performed at 10 time points after spraying, including 2, 4, 6, 8, 12, 24, 48, 72, 96, and 120 h. The expressions of *BrLRR1, BrLRR2, BrRLP47*, and *BrRLP48* in these samples were detected by qRT-PCR. The ratio of relative expression values under SA/JA treatments to those under water controls at each time point was considered to be the induced expression level after treatments. The results showed that the expression exhibited a similar wave pattern along these time points, expect *BrRLP48* in MM after SA treatment (Figure 6 and Supplementary Table S3). The expression ratios of *BrRLP48* in MM at 2, 4, and 12 h after SA treatment were 17.6, 16.5, and 61.8 respectively, which were dramatically induced compared with other genes that showed expression ratios of approximately 0–4 (Figure 6 and Supplementary Table S3). Therefore, these results suggested that *BrRLP48* was indeed induced by SA in MM and also confirmed the presence of SA-responsive elements in the promoter of *BrRLP48* in MM.

## Discussion

In this study, we identified a new QTL *Br-DM04* on Chromosome A04 for downy mildew resistance in DH and F_2_ populations derived from the cross between the Chinese cabbage line BY (*B. rapa* ssp. *pekinensis*) and a European turnip line MM (*B. rapa L.* ssp. *rapifera*). The QTLs, on Chromosome A01 and A08, for downy mildew in *Brassica rapa* reported previously were identified from different resistant sources (Yu et al., 2009, 2016; Kim et al., 2011). The minor QTL *hBrDM4* identified from resistant line T12-19, between Marker399582 and Marker 417609 (speculated physical position is between A04.2001507 and A04.3056256) on Chromosome A04, were active at the heading stage (Yu et al., 2016). The resistant QTL *Br-DM04* in this work was identified from a European turnip line MM. Although *hBrDM4* and *Br-DM04* (between A04.4285711 and A04.7136911) were both on A04, the physical locations of them implied that they are two independent QTLs. In this study, a new putative resistance gene (*R* gene), *BrRLP48*, encoding a RLP family protein was considered to confer the disease-susceptible line BY resistance toward downy mildew, in a transient expression system using *Agrobacterium* infiltration. *BrRLP47*, the expression of which was increased to about 27-fold following Agroinfiltration, could also confer resistance in BY toward downy mildew. However, the expression of *BrRLP47* was upregulated only 2.5 times at 2 h, with slight downregulation at other time points after disease infection in the resistant line MM. Therefore, *BrRLP47* could not be the crucial gene conferring resistance in MM on account of not reaching functional expression levels *in vivo* after pathogen invasion.

Agroinfiltration-mediated transient expression in *Nicotiana benthamiana* has been commonly applied to gene overexpression and silence, protein subcellular location, protein modification, interaction, and so on with its advantages of high throughput, time-saving, and high level protein production (van der Hoorn et al., 2000; Goodin et al., 2002; Koscianska et al., 2005; Liu L.J. et al., 2010). Whereas for most proteins of other plants *N. benthamiana* is a heterologous system, and there are no mutated *N. benthamiana* for gene functional analysis. In additional, the transient transformed exogenous DNA was not able to be inherited through its receptor plants, and the results were not conclusive. The transient expression in *B. rapa* improved in this work would make up the disadvantage of heterologous system, but other disadvantages still remained. Eventually, the stable genetic transformation lines should be constructed to support these preliminary findings.

Previous research demonstrated that the number of LRRs in an *R* gene for example, from the RLP and RLK families, determined the disease resistance capacity (van der Hoorn et al., 2001). *QTL-Br-DM04*, containing 17 annotated genes, was identified as a major resistant QTL with maximum LOD value 4.2, which explained up to 22.3% of the phenotypic variance. Therefore, among these genes, we chose four putative *R* genes belonging to RLP and LRR containing protein family as the candidate resistant genes. The functions of other 13 genes in *Br*-*DM04* whether or not involved in disease resistance should be further explored. Through sequence analysis, although amino acid substitutions and deletions were found in the sequence of BrRLP47 and BrRLP48 in MM compared with which in BY, the functions in disease resistance were not changed (Supplementary Figure 6). Nevertheless, the expression of *BrRLP48* was rapidly induced to high levels until 6 HAI (hours after inoculation) after which there was a slight decline at 12–24 HAI. In analogy to MM, the expression of *BrRLP48* in BY was not upregulated, but instead exhibited a mild reduction. We inferred that the elevated expression of *BrRLP48* probably contributed to the resistance to Brassica downy mildew in MM.

The functions of SA and JA as primary signals in regulating the plant’s immune response have been well described (Pieterse et al., 2009). In particular, SA is known to regulate the defense responses triggered by biotrophic pathogens which downy mildew belongs to, whereas JAs commonly control the defenses caused by pathogens with a necrotrophic lifestyle (Glazebrook, 2005). In this research, the content of SA significantly increased after pathogen invasion, whereas the content of JA decreased slightly. Even the seedlings with an overdose of SA, but not JA, could acquire resistance to downy mildew (Supplementary Figure 8). It has been directly proven that SA is involved in resistance to Brassica downy mildew, and the expression of the marker genes in SA signaling pathway was upregulated after inoculation (Gao et al., 2014). In the light of previous studies, we preferred to speculate here that *BrRLP48*, a putative R gene in downy mildew resistance, could trigger the resistant response resulting in the increment of SA after pathogen invasion. Furthermore, the upregulated expression of *BrRLP48* in response to disease could be the result of a positive feedback regulation through SA signaling. Moreover, the JA and SA responsive elements that co-exist in the promoter of *BrRLP48* would bring about the hormonal pathway crosstalk to fine-tune pathogen defense.

As one of the few identified putative *R* genes in Brassica downy mildew resistance, the disease-resistant function of *BrRLP48* should be further investigated and confirmed. Firstly, the resistance gene function of *BrRLP48* should be evaluated *in vivo* by its overexpression in transgenic disease susceptible plants. Likewise, the loss-of-function mutants of *BrRLP48* in disease resistant lines should be constructed using Clustered Regularly Interspaced Short Palindromic Repeats (CRISPR) technology, or identified the mutants of it from the EMS mutagenesis population. To confirm the important role of SA responsive element in defense response, *BrRLP48* derived by its promoter in resistant line BY with or without the SA responsive element should be transformed into the BY and the disease-resistant phenotype should be investigated. Further exploration of *BrRLP48* would assist in confirming its resistant function and understanding the possible mechanisms by which a brand new *R* gene in the RLP family of proteins mediates Brassica downy mildew resistance.

## Conclusion

Collectively, this study identified a QTL, *Bra*-*DM04*, conferring resistance to downy mildew on *B. rapa* Chromosome A04. A new *R* gene *BrRLP48* in *Bra*-*DM04*, encoding a RLP, was involved in response to Brassica downy mildew resistance and its disease responsive expression probably owing to the putative SA-responsive element in its promoter in the resistant parent only. *BrRLP48* is the most promising candidate *R* gene involving in downy mildew resistance in the European turnip line MM. The results uncover the involvement of *R* genes in Brassica downy mildew resistance and pave the way to further investigate the molecular mechanisms of disease resistance.

## Author Contributions

SY, BZ, and FZ designed the experiments. BZ, SY, TS, PL, XX, WW, YY, and DZ carried out the experiments. BZ, PL, TS, SY, and FZ wrote the paper. All authors discussed the results and commented on the manuscript.

## Conflict of Interest Statement

The authors declare that the research was conducted in the absence of any commercial or financial relationships that could be construed as a potential conflict of interest.

## Footnotes

1 http://brassicadb.org/brad/
