# Supplementary material for: BrRLP48, Encoding a Receptor-Like Protein, Involved in Downy Mildew Resistance in Brassica rapa
Source: Front Plant Sci. 2018 Nov 23;9:1708. doi: 10.3389/fpls.2018.01708 (PMC6265505; doi:10.3389/fpls.2018.01708)
Supplement: Supplementary file 2 [file Data_Sheet_1.pdf]

Supplementary Material

BrRLP48, encoding a receptor-like protein, is involved in downy mildew resistance in *Brassica rapa*

Bin Zhang*

*** Correspondence:** Shuancang Yu, yushuancang@nercv.org,
Fenglan Zhang, zhangfenglan@nercv.org

1 Supplementary Figures and Tables

1.1 Supplementary Figures

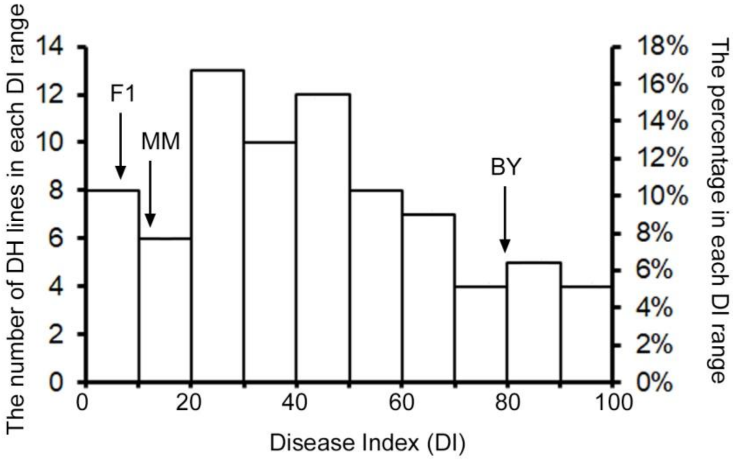

Supplementary Figure 1. The disease index of the DH population.
The arrows indicate the DI ranges which BY, MM and F1 progeny belong to.

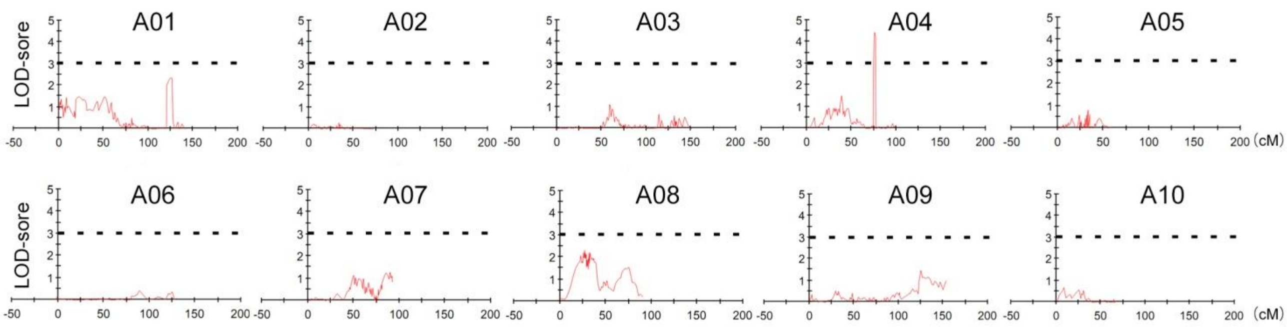

Supplementary Figure 2. QTL likelihood maps by multiple QTL model (MQM) on ten Chromosomes.
The LOD-score threshold 3 is indicated by the dashed line.

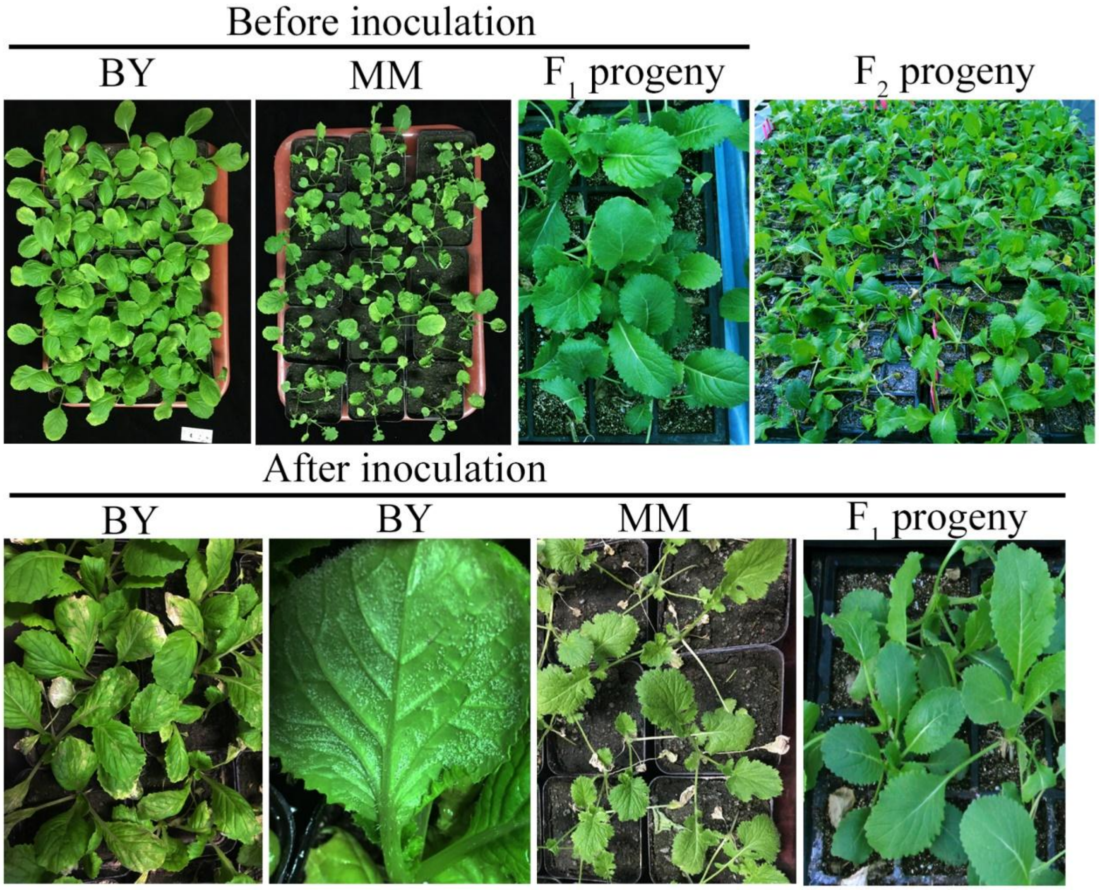

Supplementary Figure 3. The parents, F1 and F2 population used for *Bra-DM* localization. The resistant parent MM and F1 progeny showed obvious resistance to downy mildew after inoculation, while the other parent BY was susceptible.

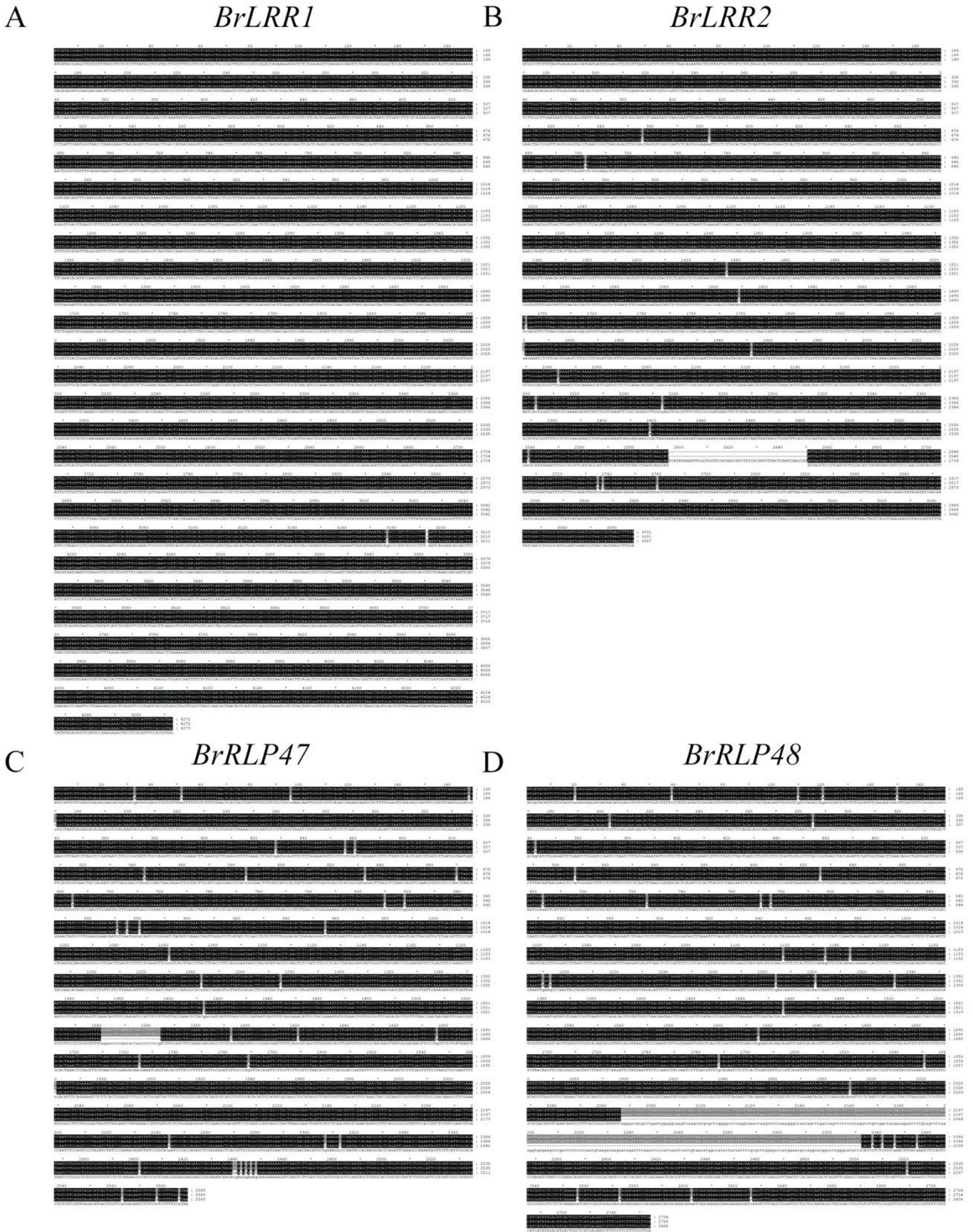

Supplementary Figure 4. The genomic sequence alignments of four candidate genes in reference, BY and MM genomes.
(A), (B), (C) and (D) are *BrLRR1*, *BrLRR2*, *BrRLP47* and *BrRLP48*, respectively. The upper, middle and lower sequences aligned are from reference, BY and MM genomes, respectively.

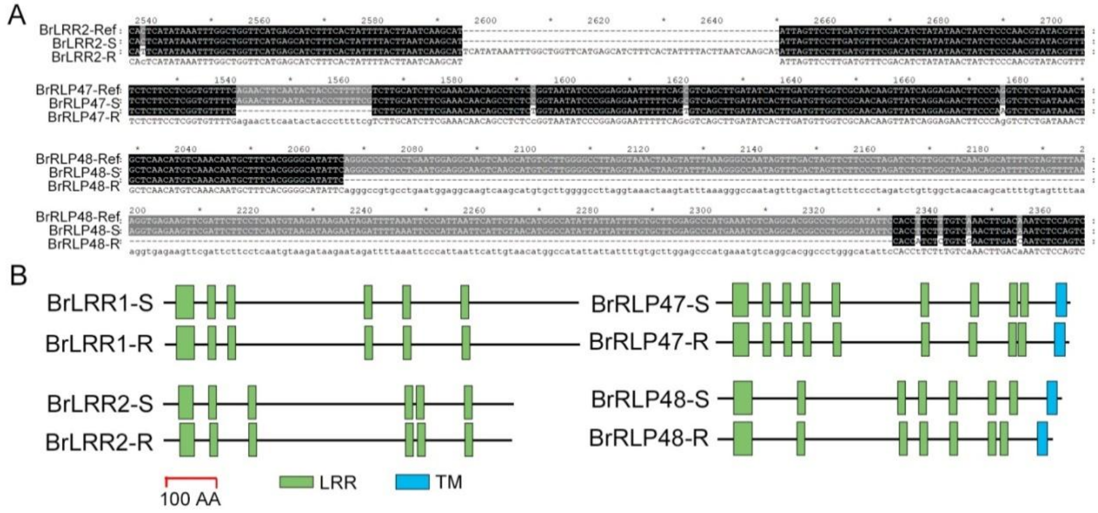

Supplementary Figure 5. Genomic sequences and protein structural analysis of candidate resistance genes in BY and MM.

(A) Large fragments of insertions and deletions exist in genomic sequences of *BrLRR2, BrRLP47* and *BrRLP48* in MM after alignment with the sequences in reference and BY genomes. (B) The protein structure predictions of four candidate genes in BY and MM. "–R" and "-S" indicate MM and BY, respectively.

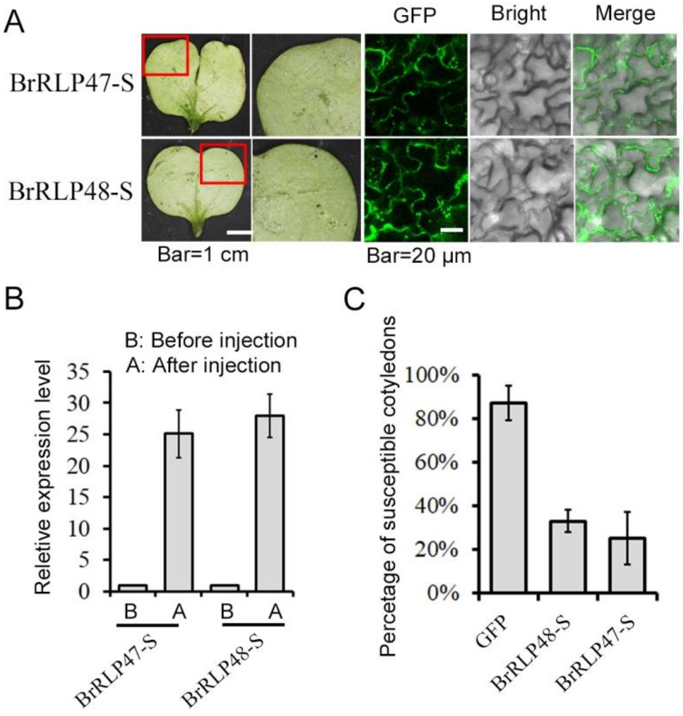

Supplementary Figure 6. Functional analysis of *BrRLP47-S* and *BrRLP48-S* (*BrRLP47* and *BrRLP48* of susceptible parent) through the agroinfiltration transient expression system.

(A) The disease resistance phenotypes and protein expression assay of cotyledons injected with *BrRLP47-S* and *BrRLP48-S* were observed three days after agroinfiltration. The images in red squares are magnified in the pictures in the second column from the left. (B) Gene expression analysis after agroinfiltration. Expression of each gene before injection was defined as 1.0. Values represent means ±SD (n = 3) from three technical replicates. (C) The percentage of susceptible

injected cotyledons after inoculation. Values represent means ±SD from three independent experiments. Each experiment tested at least 50 cotyledons.

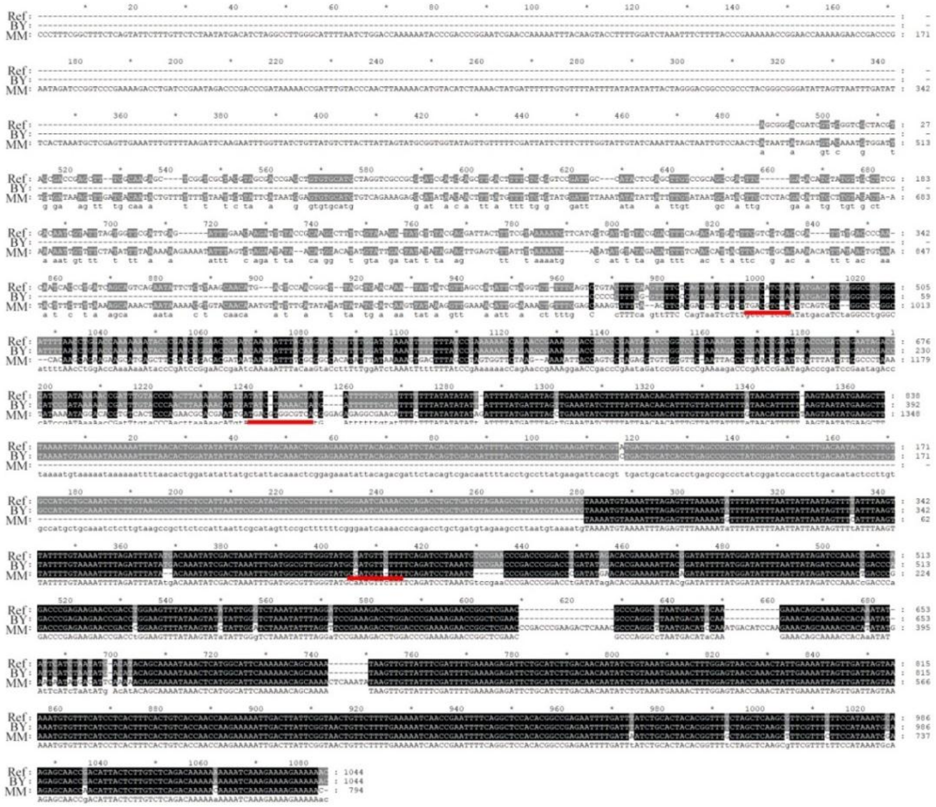

Supplementary Figure 7. The promoter sequence alignments of *BrRLP48* in reference, BY and MM genomes.
The red lines below the alignments indicate the predicted transcript elements in MM.

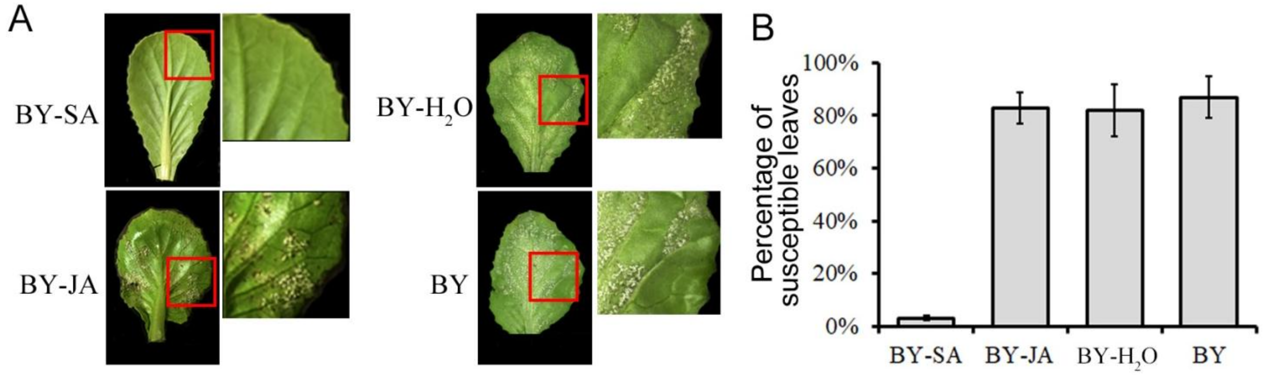

Supplementary Figure 8. Disease resistant of SA and JA treated seedlings after downy mildew inoculation in susceptible line BY
(A).The investigation to downy mildew in BY leaves after SA, JA and H_2O spraying treatment. The imagines in red squares were magnified and placed on the right. (B). The percentages of susceptible leaves in different treatments. Values represent means ±SD from three independent experiments. Each experiment tested at least 50 cotyledons.

1.1 Supplementary Tables

The Supplementary Tables for this article can be found in the attached file with excel format.
